# Kinetics of Regenerating Protein 3E and C‐Reactive Protein in Healthy Dogs, Dogs Undergoing Tibial Plateau Leveling Osteotomy, and Dogs With Sepsis

**DOI:** 10.1111/vcp.70115

**Published:** 2026-06-12

**Authors:** Laura Gentilini, Johanna Rompf, Judith Howard, Simone Schuller, Laureen M. Peters

**Affiliations:** ^1^ Clinical Diagnostic Laboratory, Department of Clinical Veterinary Science, Vetsuisse Faculty University of Bern Bern Switzerland; ^2^ Division of Small Animal Emergency and Critical Care, Department of Clinical Veterinary Science, Vetsuisse Faculty University of Bern Bern Switzerland; ^3^ Division of Small Animal Internal Medicine, Department of Clinical Veterinary Science, Vetsuisse Faculty University of Bern Bern Switzerland

**Keywords:** acute‐phase protein, infection, inflammation, pancreatitis‐associated protein, surgical trauma

## Abstract

**Background:**

Serial measurements of acute‐phase proteins help monitor the development of and recovery from sepsis in people and dogs. To date, the kinetics of regenerating protein (REG) 3E, a novel biomarker for inflammation in dogs, are unknown.

**Objectives:**

The goal of our study was to assess changes in the concentrations of REG3E and compare these to C‐reactive protein (CRP) concentrations over 48–72 h in different cohorts of dogs.

**Methods:**

Plasma REG3E concentrations were measured using an ELISA on 3–4 consecutive days from 43 dogs, including 12 healthy controls, 17 undergoing tibial plateau leveling osteotomy (TPLO), and 14 with sepsis. Canine CRP was measured using a turbidimetric immunoassay.

**Results:**

REG3E concentrations were low in control and TPLO dogs throughout all time points. In the TPLO group, there was a mild but significant increase in CRP over time. REG3E and CRP concentrations were higher in septic dogs at admission compared to the other groups and progressively decreased after initiation of treatment to 14% and 28% of initial levels, respectively, within 72 h. REG3E, but not CRP concentrations, stayed high in two dogs, one of which developed recurrent sepsis. There was a significant moderate correlation between both acute‐phase proteins among all groups and within the sepsis group, but not within the TPLO group.

**Conclusions:**

In conclusion, based on this cohort of dogs, CRP appears to be more sensitive to detect minor postsurgical inflammation, but the potential of REG3E to predict developing or recurrent sepsis deserves further investigation.

## 1 Introduction

Sepsis, a major global cause of morbidity and mortality in people, is defined as an overzealous response of the host to an infectious process, potentially triggering life‐threatening organ dysfunction [1]. Similarly, in dogs, sepsis, for example, due to pneumonia, peritonitis, and pyometra, is associated with high mortality [2, 3]. The effectiveness of interventions to improve chances of survival in septic patients, particularly the administration of antimicrobial treatment, relies on their timely application, which is linked to early and reliable diagnosis [4, 5]. Unfortunately, confirmation of sepsis, particularly differentiation from noninfectious systemic inflammatory response syndrome (SIRS), in dogs is challenging, in part due to their similar clinical presentations and lack of specific and rapid diagnostic tests [2, 6].

C‐reactive protein (CRP) is currently the most widely used inflammatory marker in canine medicine, which has proven to be highly sensitive but not specific for inflammation and infection [7, 8]. It is considered a major positive acute‐phase protein due to its marked and rapid increase, detectable as soon as 4 h after inflammatory stimulation, and peaking at 24–48 h [9]. Procalcitonin (PCT), an inflammatory marker more recently gaining popularity in human medicine, was shown to increase in healthy research dogs 2 h after lipopolysaccharide injection, peaking after 4 h, and returning to baseline concentrations within 48 h [10]. Despite promising performance as a diagnostic and prognostic marker for sepsis in people, PCT has not proven to be reliable in dogs, as it does not consistently increase in the face of confirmed bacterial infection [4, 11]. Therefore, the search for more dependable biomarkers is ongoing.

In people, pancreatic stone protein, also known as regenerating protein or regenerating island‐derived protein (REG) 1A, a C‐type lectin predominantly produced in the pancreas [12], shows promise as an emerging biomarker for sepsis, both for early diagnosis and prognosis [13, 14]. For example, REG1A concentrations increased significantly in people with severe trauma and burns developing infection 2–3 days before clinical manifestation of sepsis [13, 15]. A member of this protein family, REG3E, a homolog to human REG3A also called pancreatitis‐associated protein, was recently discovered in dogs [16] with very high concentrations in sepsis but also in pancreatitis compared to healthy controls, making it a positive acute‐phase protein with potential as an inflammatory marker in this species [17]. Yet to date, very little is known about the function, origin, and kinetic properties of REG3E.

The aims of this study were to describe the plasma kinetics of REG3E and CRP concentrations in the first days of hospitalization in dogs with sepsis and compare these to concentrations in dogs undergoing elective orthopedic surgery and in clinically healthy dogs. We hypothesize that REG3E concentrations decrease rapidly after resolution of sepsis, that orthopedic surgery only leads to a mild increase in REG3E concentrations, and that day‐to‐day fluctuations are negligible in clinically healthy dogs.

## 2 Materials and Methods

### 2.1 Samples and Animals

The majority of samples used in this observational study were leftover aliquots from dogs prospectively enrolled as part of two other observational studies, approved by the local animal welfare committee (Ref. BE 127/19), at the Small Animal Clinic of the Vetsuisse Faculty, University of Bern, Switzerland, between June 2020 and March 2021 [11, 18]. Some samples were retrospectively included, leftover convenience samples from routine diagnostic submissions to our diagnostic laboratory from client‐owned dogs with naturally occurring sepsis, hospitalized between January 2023 and March 2024. For all samples, signed owner consent for the use of clinical data and leftover biological material was obtained. Laboratory handling was the same for all samples: Lithium‐heparinized whole blood was centrifuged for 10 min at 3000 *g* (Mikro 200; Hettich AG, Bäch, Switzerland), and the supernatant plasma was aliquoted and stored at −80°C until the time of acute‐phase protein analysis.

#### 2.1.1 Healthy Group

The healthy group included dogs owned by staff and students from our institution or the Swiss army. They were considered clinically healthy based on an unremarkable history, physical examination findings, and routine hematological and biochemical results, including CRP concentrations within the reference interval (RI; < 10.2 mg/L) on the day of the first sample. In total, healthy dogs were sampled on three consecutive days (D0, D1, D2).

#### 2.1.2 TPLO Group

Dogs in the tibial plateau leveling osteotomy (TPLO) group were client‐owned patients of the Small Animal Clinic of the Vetsuisse Faculty, University of Bern, Switzerland, diagnosed with cranial cruciate ligament (CCL) rupture, which underwent routine general anesthesia, stifle joint arthroscopy, and TPLO surgery. Details about the diagnosis and treatment of this cohort can be found in the parent study [18]. Blood was sampled on Day 0 (D0; preoperative), Day 1 (D1; immediately after surgery), and Day 2 (D2; 1 day post‐surgery).

#### 2.1.3 Sepsis Group

Dogs in the sepsis group were client‐owned patients of the Small Animal Clinic of the Vetsuisse Faculty, University of Bern, Switzerland. They were enrolled in this cohort if bacterial infection was confirmed by direct identification of bacteria in cytological or histopathological samples, bacterial culture, or intraoperative detection of pus, and if they had evidence of systemic inflammatory response syndrome (SIRS), based on the clinical criteria established by Hauptman et al. [19] Specifically, dogs met SIRS criteria if they had at least two of the following characteristics at the time of admission: body temperature < 38.1°C or > 39.2°C; heart rate > 120 beats per minute; respiratory rate > 20 breaths per minute; white blood cell count (WBC) < 6.0 × 10^9^/L or > 16.0 × 10^9^/L or > 3% band neutrophils, determined by a manual differential count. Blood was sampled at admission (D0) and on Day 1 (D1) in all cases, and on Day 2 (D2) and/or 3 (D3) post admission, depending on clinical necessity and weekday. Where surgical interventions were necessary for the treatment of the infection, these were performed between sampling Day 0 and Day 1. All dogs received antimicrobial drugs as part of their treatment, some of which were prescribed prior to presentation by the referring veterinarian.

### 2.2 Laboratory Testing

All laboratory analyses were performed in the Clinical Diagnostic Laboratory, Vetsuisse Faculty, University of Bern, Switzerland. Routine hematologic and biochemical testing was carried out on the ADVIA 2120i (Siemens Healthcare, Zurich, Switzerland) and the Cobas c501 (Roche Diagnostics, Basel, Switzerland), respectively, on the day of sampling or the subsequent weekday for out‐of‐hours samples. Concentrations of CRP were measured batch‐wise using a previously validated canine‐specific immunoturbidimetric assay (Gentian canine CRP, Gentian AS, Moss, Norway [20]) after a maximum storage period of 18 months. Concentrations of REG3E were measured with a validated sandwich ELISA [21] using an automated plate washer and reader (Crocodile 5‐in‐one ELISA miniWorkstation; Berthold Technologies GmbH & Co. KG, Bad Wildbach, Germany). Briefly, this assay uses two custom‐made, purified polyclonal anti‐canine REG3E antibodies, raised in guinea pigs and rabbits, as well as a tertiary anti‐rabbit IgG antibody (Sigma‐Aldrich, Buchs, Switzerland) coupled with alkaline phosphatase. Standards, pooled quality control samples (3 levels), and canine plasma samples were run in duplicates, diluted at 1:25, 1:250, 1:1000, 1:2000 or 1:5000 (in 1% bovine serum albumin in tris‐buffered saline) to fall within the measuring range of 30–300 ng/mL. This assay underwent thorough analytical validation, including for use of both serum and heparinized plasma, and demonstrated robust sample stability [21]. Results below the lower limit of quantification (LLoQ) were recorded as < 30 ng/mL and set as 30 ng/mL for statistical analyses. Coefficients of variation (CV) were calculated as specified below between duplicate measurements of all samples on the same plate and between controls on different plates.

### 2.3 Statistical Analysis

Statistical analysis was performed using MedCalc Statistical Software (version 23.0.2; MedCalc Software Ltd., Ostend, Belgium) unless otherwise stated, and results were considered to be statistically significant if *p* ≤ 0.05. Kruskal–Wallis test was used to calculate differences in age between groups, and Fisher's exact test (https://astatsa.com/; accessed 14.1.2025) was used to evaluate differences in sex distribution between groups. Concentrations of REG3E and CRP were compared between groups using Kruskal–Wallis tests with post hoc Conover analysis, where *p* was set at < 0.01 to account for multiple testing. Within the healthy group, day‐to‐day variability and interindividual variability of REG3E concentrations were assessed by calculation of CVs (standard deviation/mean × 100) expressed in %. Differences between REG3E and CRP concentrations, respectively, at different time points were calculated with the Friedman Test for groups with complete data sets (healthy and TPLO groups) with a Bonferroni‐corrected *p*‐value of < 0.01. A mixed effects model (Graphpad Prism Software version 10.4.1, Mosten MA, USA) was used in the septic group for the trajectory of both proteins over time, to account for cases with missing values on Days 2 or 3. Additionally, the percentage decrease in REG3E and CRP concentrations compared to baseline values was calculated using the following formula: [protein]_Day1_/[protein]_Day0_ × 100. Measurements recorded to be < 30 ng/mL for REG3E and < 6.4 mg/L for CRP (LLoQ), respectively, were excluded from this analysis. Spearman's rank correlation coefficient was used to assess the relationship between REG3E and CRP concentrations overall and within different study groups.

## 3 Results

### 3.1 Demographic Data

A total of 43 dogs were included, including 12 in the healthy group, 17 in the TPLO group, and 14 in the sepsis group (Table 1). There was no significant difference in age between the groups (*p* = 0.19). There was a significant difference in sex between the groups (*p* = 0.02), with a female predominance in the sepsis group (64%). The majority of males in the healthy group were entire, whereas no entire males were recorded in the TPLO and sepsis groups; in the TPLO group, most females were spayed.

**TABLE 1:** Demographic data and acute‐phase protein concentrations of 43 healthy dogs with no clinical disease, dogs undergoing TPLO for cranial cruciate ligament rupture, and dogs with bacterial sepsis. *p*‐values indicate significant differences among groups (Kruskal–Wallis test for continuous variables; Fisher's exact test for categorical variables).

Variable	Healthy	Healthy after exclusion of military dogs	TPLO	Sepsis	*p*
*n*	12	7	17	14	
Age (years), median (min; IQR; max)	3.5 (1; 2.7–7.9; 11.7)	6.2 (1; 4.4–10.9; 11.7)	5.7 (0.7;3.8–7.5;13.5)	8.4 (1.7; 3.3–9.7;11.1)	0.19
Sex, *n* (%)					
Male; neutered	6 (50%); 2	3 (43%); 2	8 (47%); 8	5 (36%); 5	0.02
Female; spayed	6 (50%); 3	4 (57%); 2	9 (53%); 8	9 (64%); 4	
REG3E (D0) (ng/mL), median (min; IQR; max)	61 (< 30; < 30–212.5; 335)	30 (< 30; < 30–42; 191)	42 (< 30; < 30–77.8; 300)	1361.5 (250; 498–2290.3; 10 698)	< 0.01
REG3E (D1) (ng/mL), median (min; IQR; max)	96 (< 30; 44–210; 320)	46 (< 30; 33–80.5; 275)	32 (< 30; < 30–52.3; 182)	864.5 (94; 479–3191; 12 985)	< 0.01
REG3E (D2) (ng/mL), median (min; IQR; max)	95 (< 30; 33.5–245.5; 319)	36 (< 30; 30.3–55.8; 296)	< 30 (< 30; < 30–39; 46)	376 (56; 176–975; 2833) *n* = 10	< 0.01
REG3E (D3) (ng/mL), median (min; IQR; max)	—		—	175.5 (43; 95–928; 13 723) *n* = 10	—
CRP (D0) (mg/L), median (min; IQR; max)	All values < 6.4	All values < 6.4	< 6.4 (< 6.4; < 6.4–< 6.4; 8.7)	160.9 (55.7; 118.2–211.4; 402.9)	< 0.01
CRP (D1) (mg/L), median (min; IQR; max)	All values < 6.4	All values < 6.4	< 6.4 (< 6.4; < 6.4–11; 31.5)	108.1 (23.3; 82.7–167.3; 260.3)	< 0.01
CRP (D2) (mg/L), median (min; IQR; max)	All values < 6.4	All values < 6.4	25.3 (9.4; 19.3–42.3; 109.7)	61.4 (10.5; 32.9–96.2; 102.9) *n* = 10	< 0.01
CRP (D3) (mg/L), median (min; IQR; max)	—		—	67.6 (31.5; 47–89.8; 128.6) *n* = 9	—

Abbreviations: CRP, C‐reactive protein; D0, Day 0; D1, Day 1; D2, Day 2; D3, Day 3; IQR, interquartile range; REG3E, regenerating protein 3E; TPLO, tibial plateau leveling osteotomy.

The majority of dogs were medium to large breeds, with mixed‐breed dogs predominating overall (7/43; 16.3%). In the healthy group, Shepherd breeds were overrepresented (3 Belgian Malinois, 2 Belgian Tervueren, 2 German Shepherds, 1 Dutch Shepherd), 5 of which were enrolled in the military service. The TPLO group included three Labrador Retrievers, two Bernese Mountain dogs, and one dog each from other breeds. In the sepsis group, mixed‐breed dogs predominated (*n* = 4), with two each of American Staffordshire Terrier, Border Collie, and Labrador Retriever, and one dog each from other breeds.

### 3.2 REG3E Kinetics

Concentrations and distribution of REG3E at different time points are listed for each group and sampling time point separately in Table 1 and illustrated in Figure 1A–C. There was no significant difference in REG3E concentrations between the three time points in healthy dogs (*p* = 0.24). After exclusion of REG values recorded as < 30 ng/mL, the mean day‐to‐day intraindividual variability was 24% (range 4%–68%) and the mean interindividual variability of all 3 days was 64% (range 53%–71%). It is noteworthy that REG3E concentrations were significantly higher in military working dogs (dogs H7–H11 in Figure 1A) throughout all time points (*p* < 0.05; median, D0 228; D1 201; D2 215 ng/mL) than in pet dogs (median, D0 30; D1 46; D2 36 ng/mL), and that the interindividual variability was considerably lower in this sub‐cohort (mean; 39%).

**FIGURE 1:**
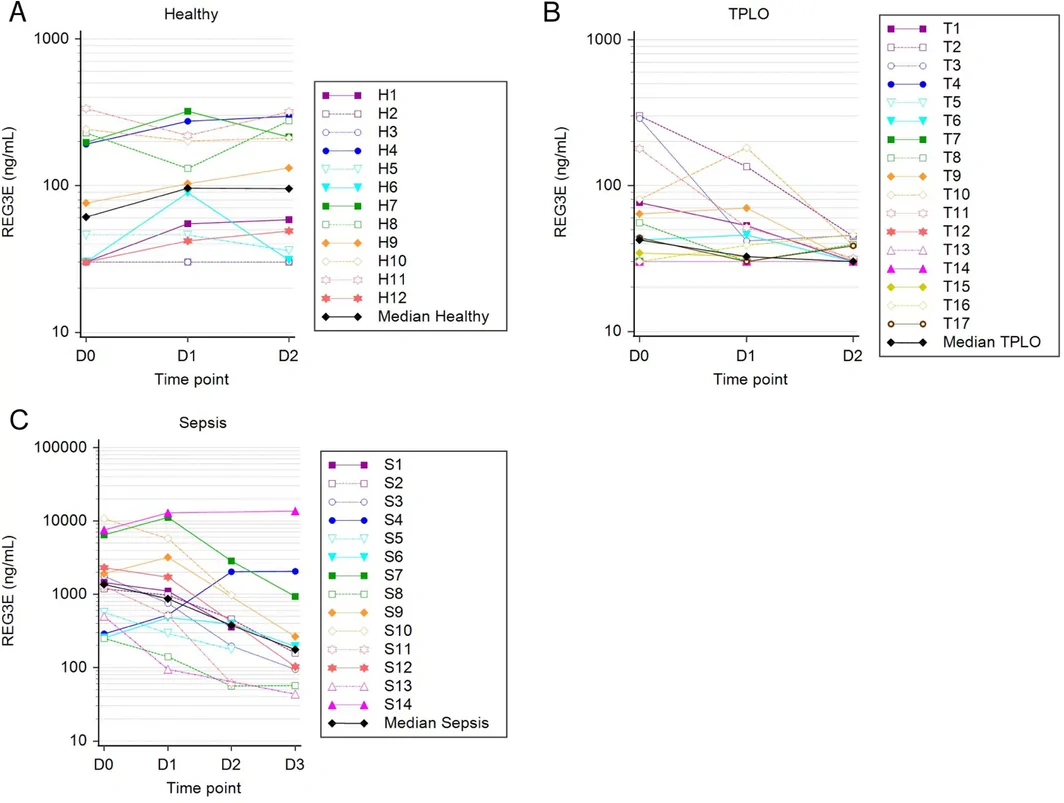
Kinetics of regenerating protein 3E (REG3E) concentrations (log transformed) over three to four consecutive days in healthy dogs (A), in dogs undergoing tibial plateau leveling osteotomy (TPLO) (B), and in dogs with sepsis (C). The median is depicted as a solid black line.

There was a progressive, mild, but nonsignificant decrease in REG3E concentrations over time in dogs undergoing TPLO (*p* = 0.03). Overall, REG3E concentrations decreased in seven cases from D0 to D1 (median overall decrease to 70% of baseline activity) and in 10 cases from D0 to D2 (median overall decrease to 49%). Only one case had a net increase in REG3E concentration over time, with D2 being 153% that of D0, but staying very low overall (max. 46 ng/mL on D2). In one dog, REG3E concentration peaked at D1 (182 ng/mL, or 227% of baseline), but on D2, the concentration returned to 50% of that of D0.

Among the 14 dogs with sepsis, nine had a septic abdomen, three had pyometra, one had a neck abscess after esophageal perforation, and one had pyelonephritis. The main causes of septic abdomen were enterotomy suture dehiscence, perforation of the gastrointestinal tract, and prior surgical excision of intra‐abdominal lymph nodes or tumors. REG3E concentrations were measured in all 14 dogs at D0 and D1, and in 10 each at D2 and D3; six dogs had measurements at all time points. Overall, there was no significant difference in REG3E concentrations between different time points in the sepsis group (*p* = 0.22), although there was a net median decrease in REG3E concentrations on D1 to 75%, on D2 to 28%, and on D3 to 14% from baseline. Concentrations increased in five cases from D0 to D1, and in two cases from D0 to D2, with an increase to 706% (2027 ng/mL) in one case, which remained similarly high at D3 (2096 ng/mL), despite clinical and hematological improvement of inflammation (Case S4 in Figure 1C). This patient was initially treated for a septic abdomen after suture dehiscence and postoperatively developed acute kidney injury, with creatinine concentrations peaking at D2 (599 μmol/L, RI: 52–117), and concurrently had hyperlipasemia, with DGGR‐lipase activity on D2 of 1803 U/L (RI: 24–108) and peaking on D3 (4292 U/L). In the second case, REG3E concentrations increased progressively at all time points, almost 200% from baseline to D3 (D0: 7589 ng/mL; D1: 12 985 ng/mL; D3: 13 723 ng/mL; Case S14 in Figure 1C). This dog initially underwent abdominal surgery for enterectomy and end‐to‐end anastomosis on D0, but had a recurrence of a septic abdomen documented 1 week after the initial diagnosis, upon which it was euthanized due to poor prognosis. This was the only non‐survivor (to discharge) in the entire study.

Comparing REG3E concentrations between the different groups at different time points revealed significantly higher concentrations in the sepsis group at D0 (*p* < 0.01; Table 1), which remained significant through D1 and D2. On D1 and D2, healthy dogs also had significantly higher REG3E concentrations compared with TPLO dogs (*p* < 0.01; Table 1). This was, however, no longer the case after exclusion of military working dogs from the healthy group (*p* > 0.01).

### 3.3 CRP Kinetics

Concentrations of CRP at different time points in different groups are listed in Table 1 and illustrated in Figure 2A,B. As CRP was below the LLoQ (< 6.4 mg/L) in all healthy dogs at all time points, kinetics and day‐to‐day variability could not be calculated for this parameter in this group.

**FIGURE 2:**
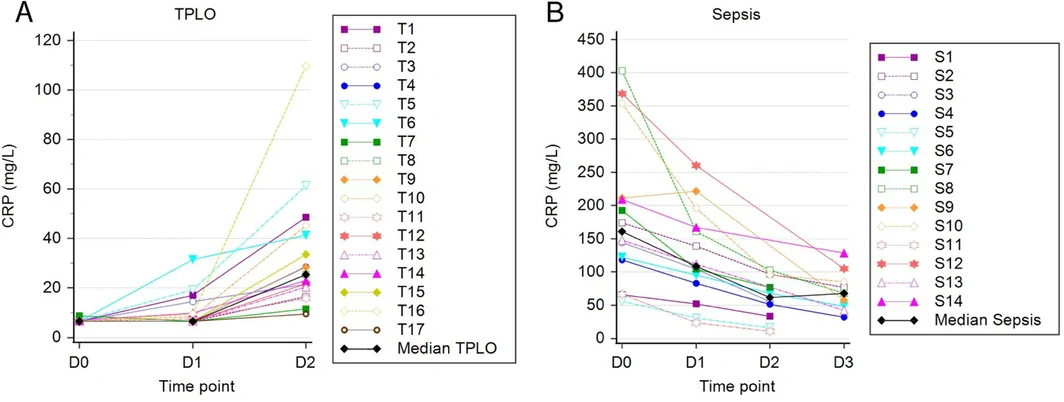
Kinetics of C‐reactive protein (CRP) concentrations over three to four consecutive days in dogs undergoing tibial plateau leveling osteotomy (TPLO) (A) and in dogs with sepsis (B). The median is depicted as a solid black line.

All but one case had measurements available for CRP at all time points. Within the TPLO group, there was a significant progressive increase in CRP over time (*p* < 0.01). Concentrations increased in eight cases from D0 to D1 (median increase to 148%) and in all cases from D0 to D2 (median increase to 395% or 25.3 mg/L).

Measurements of CRP were available for all dogs of the sepsis group at D0 and D1, for 10 dogs at D2, and 9 at D3; 5 dogs had measurements at all four time points. There was a significant progressive decrease in CRP concentrations over time (*p <* 0.01). CRP concentrations decreased in 13/14 cases from D0 to D1 and in all cases from D0 to D2 and D3. A median decrease was found from baseline to D1 to 72%, to D2 to 39%, and to D3 to 28%. In cases S4 and S14, in which REG3E did not decrease over time, CRP did indeed decrease noticeably (Case S4, Figure 2B, D0: 118.2 mg/L; D1: 82.7 mg/L; D2: 50.8 mg/L; D3: 31.5 mg/L. Case S14, Figure 2B, D0: 209.3 mg/L; D1: 167.3 mg/L; D3: 128.6 mg/L).

Concentrations of CRP were significantly higher in dogs with sepsis compared to healthy dogs at all time points, and higher than TPLO dogs on D0 and D1 (*p <* 0.01), but not on D2. Additionally, CRP concentrations were greater in TPLO dogs compared with healthy controls on D1 and D2 (*p <* 0.01), but not on D0 (Table 1).

### 3.4 Correlation Between REG3E and CRP

Among all samples of all groups, including all time points (*n* = 134), there was a moderate correlation between REG3E and CRP concentrations (*r*_s_(65) = 0.5; 95% CI: 0.36–0.62; *p* < 0.01) (Figure 3A). There was no correlation for samples within the TPLO group (*p* = 0.19), whereas within the septic group, there was a moderate correlation (*r*_s_(21) = 0.44; 95% CI: 0.17–0.65; *p* < 0.01) (Figure 3B).

**FIGURE 3:**
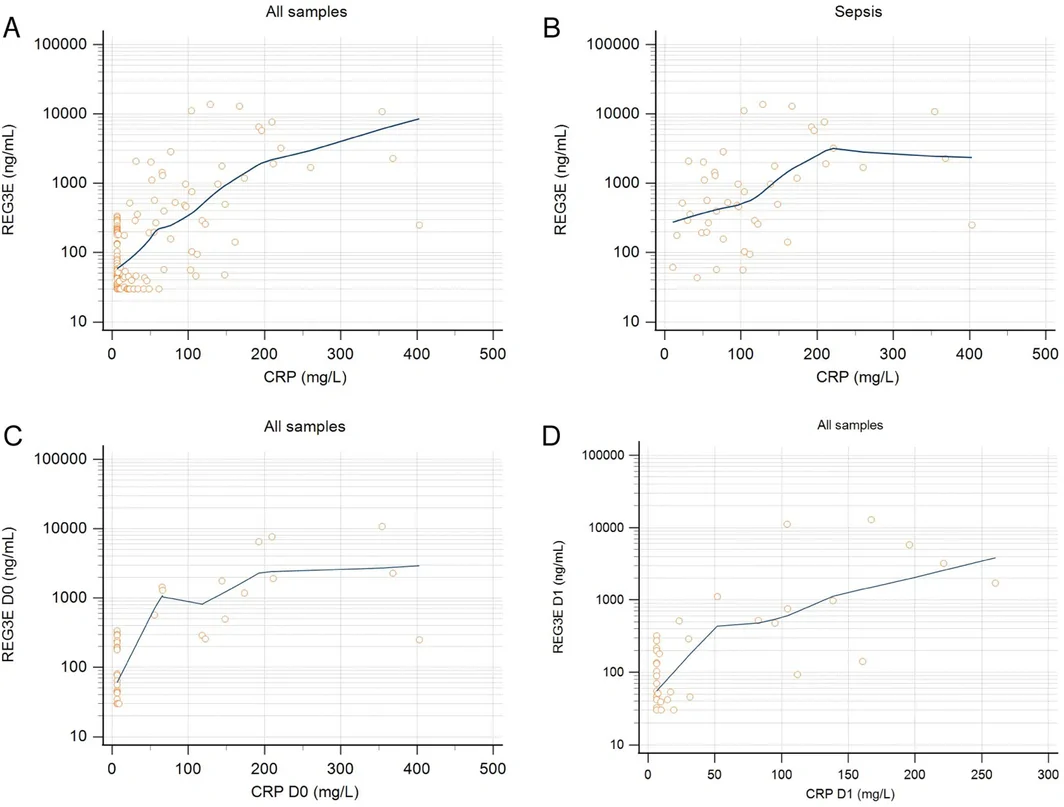
Rank correlation between regenerating protein 3E (REG3E; log transformed) and C‐reactive protein (CRP) concentrations in dogs, including all groups at all time points (A), only the sepsis group at all time points (B), all groups on Day 0 (C), and all groups on Day 1 (D).

When split into different sampling time points, the correlation between REG3E and CRP across all groups was strongest on D0 (*r*_s_(19) = 0.67; 95% CI: 0.46–0.81; *p* < 0.01) and D1 (*r*_s_(21) = 0.63; 95% CI: 0.41–0.78; *p* < 0.01; Figure 3C,D), but there was no correlation on D2 (*p* = 0.54) nor on D3 (*p* = 0.78). Within the septic group, there was no correlation between both analytes at any individual time point (all *p* > 0.19).

## 4 Discussion

In healthy dogs, REG3E concentrations were low at all three time points, as expected in the absence of inflammation or interventions, with no significant difference between time points. We calculated a higher interindividual variability of REG3E (64%) than intraindividual (day‐to‐day) variability (24%) in healthy dogs, paralleling observations of other acute‐phase proteins in this species. Concretely, in the same cohort of healthy dogs, the interindividual variability of PCT (36%) was greater than the intraindividual variability (15%) [18]. Similarly, canine CRP was reported to have a greater interindividual variability (17%) than intraindividual variability (1%) [22], but this could not be investigated in our cohort due to all healthy dogs having CRP concentrations below the LLoQ. It is suspected that the large interindividual variability reflects physiologic differences between pet dogs and working dogs enrolled in our healthy cohort, as the healthy military subgroup had significantly higher REG concentrations than the healthy pet dogs. Lower intraindividual variability suggests that it is more reliable to compare serial measurements of REG3E from one dog, than to interpret REG3E concentrations alongside population‐based reference intervals. Particularly when population‐based reference intervals are wide, changes in laboratory parameters of a single individual may be missed if they remain within normal limits [23].

In the TPLO group, we would have expected a mild increase in REG3E concentrations post‐surgery, paralleling our observations for CRP, but instead, we saw a mild progressive but statistically insignificant decrease in REG3E over time. The most plausible explanations for this lack of increase are that either the inflammatory stimulus through surgery was too minor to increase REG3E expression, or that an increase in REG3E takes longer than 48 h to develop and was thus missed in our study. Furthermore, hemodilution by peri‐ and intraoperative fluid administration may have masked a peak in REG3E concentrations, but this is difficult to evaluate in a surgical scenario with concurrent hemorrhage also influencing concentrations of proteins and erythrocytes, and, less plausibly, as such a dilutional effect should have also decreased CRP concentrations, which, on the contrary, increased slightly. Similarly, the study investigating PCT in the same cohort of TPLO dogs found a mild postoperative decrease, rather than an increase in PCT [18].

In contrast, CRP concentrations increased progressively over the first 48 h post TPLO surgery in our study cohort. A CRP peak at 24 h of approximately 100 mg/L was also described in dogs undergoing TPLO in another study, where CRP normalized within 6 days (median, 20.6 mg/L) in dogs without postoperative complications. In dogs experiencing complications such as local infection or noninfectious lesions like meniscal tears, tibial crest fractures, and seroma formation, CRP concentrations remained high with a median value of 92.9 mg/L and only normalized by the 12th day [24]. Similar kinetics were also described for dogs undergoing other orthopedic surgeries, such as patella luxation, with a significant median increase within 24 h of almost 100 mg/L, followed by a minimal decrease within 48 h after surgery [25]. The magnitude of the CRP peak 24 h postoperatively in these studies is considerably higher than in ours, which may mirror different surgical procedures, as well as the degree of surgical trauma and inflammation, but also different CRP assays used.

Although orthopedic surgeries like TPLO are not directly comparable with laparotomies, there is support in the literature that the kinetics of acute‐phase proteins behave similarly with both interventions. In particular, in healthy research dogs undergoing elective orchidectomy, a 17‐fold increase in CRP (to a mean of approximately 100 mg/L) was observed in the first 24 h postoperatively, followed by a rapid decline within 48 h [26]. In clinically healthy bitches undergoing elective ovariohysterectomy (OHE), CRP peaked in the first 24 h after surgery to a mean of 104 mg/L, and decreased only slowly, staying significantly increased throughout 10–17 days postoperatively [27]. Another study measuring CRP every 4 h after vasectomy, laparoscopic OHE, and laparotomic OHE found that CRP increased sharply between 4 and 12 h and peaked at 24 h, with mean CRP concentrations highest in the laparotomic OHE group, reaching approximately 30 mg/L [28]. This magnitude of increase is more comparable with the postoperative increase in our TPLO group, suggesting the magnitude of inflammation in both these non‐septic surgeries may be similar. This allows the conclusion that the laparotomy itself likely only contributes minimally to the inflammatory stimulus in those dogs in our septic group undergoing abdominal surgery as part of their treatment.

As expected from a previous study [17], we saw significantly and markedly (20‐ to 30‐fold) higher REG3E concentrations at D0 in dogs with sepsis compared to the healthy and the TPLO groups, largely exceeding our calculated interindividual variability. Although overall REG3E concentrations decreased rapidly in most dogs of this group, concentrations increased in the first 24 h in five cases, making the decrease overall statistically insignificant. Upon detailed examination of the individual cases, we concluded that this was likely mainly attributable to two dogs, in which REG3E concentrations continued to increase despite initiation of treatment for sepsis. One dog, in which REG3E increased markedly on D1 and stayed high on D3, experienced a recurrence of septic peritonitis diagnosed 1 week after the initial presentation for septic peritonitis. In contrast, CRP concentrations decreased over time in this dog. These findings suggest that REG3E remained high due to ongoing/recurrent sepsis and that this protein may potentially be useful to predict such events. In humans, it has been widely demonstrated that REG1A is an earlier and more reliable predictor for the development of sepsis compared with other acute‐phase proteins. In one study on early detection of nosocomial sepsis in patients hospitalized in the intensive care unit, an increase in concentrations of REG1A was detected 2 days earlier than that of PCT, 3 days earlier than that of CRP, and 5 days before the clinical diagnosis of sepsis [29]. In a study on sepsis detection in burn patients, REG1A demonstrated a threefold increase 3 days before, and a twofold increase 2 days before manifestation of clinical signs [13]. The second dog with increasing REG3E concentrations during hospitalization had relatively low REG3E concentrations at presentation with septic peritonitis, which increased several 100‐fold from baseline by D2 and remained similarly high despite clinical and hematological improvement of inflammation, with CRP concentrations decreasing over time. Postoperatively, this dog developed acute kidney injury and suspected concurrent pancreatitis based on an important increase in DGGR‐lipase activity. Pancreatitis is well known to lead to markedly increased REG1 and REG3 concentrations in humans, hence REG3A also being called “pancreatitis‐associated protein” [30, 31]. In dogs, one study so far has documented significantly higher REG3E concentrations in acute pancreatitis with levels similarly high as in sepsis, leading to the conclusion that REG3E concentrations could likely not distinguish between sepsis and acute pancreatitis [17]. Furthermore, both REG1A and REG3A have been shown to be markedly increased in humans with kidney disease [32, 33], but this has to date not been explored in dogs. Taken together, we suspect that both renal and pancreatic disease may have led to a sustained increase in REG3E concentration in this dog despite clinical resolution of sepsis, but more information on REG3E expression, metabolism and excretion is needed to allow for a deeper understanding of the significance of increased REG proteins in this species.

In contrast to REG3E, the decrease in CRP over time was significant in our sepsis group, paralleling the clinical improvement of sepsis and inflammation in all but one case. This is in keeping with previous studies describing CRP kinetics in dogs undergoing emergency laparotomy for gastric dilatation volvulus or pyometra. In dogs with gastric dilatation volvulus, CRP concentration was low before and immediately after surgery, peaked at 24 h postoperatively to a median of 128 mg/L, and decreased slightly by 48 h (median: 116 mg/L) [34]. Comparing bitches undergoing elective OHE with those undergoing OHE for pyometra, the latter had higher mean CRP concentration preoperatively (healthy: 2.8 mg/L; pyometra: 86.7 mg/L), peaking 24 h postoperatively in both groups (healthy: mean 103.8 mg/L; pyometra: mean 156.7 mg/L), but declining faster in dogs with pyometra from the 3rd day onwards (healthy: mean 81.0 mg/L; pyometra: mean 58.9 mg/L) [27]. In another study comparing CRP concentrations in dogs with and without complications after OHE for pyometra, CRP remained high in dogs with complications, such as inflammation of the surgical wound and surrounding tissues on Day 3 after surgery (as opposed to a significant decrease of CRP in dogs without complications) and even mildly increased on Day 4, the day when the complication was clinically manifest [35]. Comparison of serial measurements of CRP and REG3E over a longer period of time in dogs with and without postoperative complications is warranted to further assess the ability of these acute‐phase proteins to reflect and predict ongoing and developing inflammation and infection.

Despite these observed discrepancies between REG3E and CRP, both in the TPLO and the sepsis group, we found a moderate correlation between both acute‐phase proteins among all groups and within the sepsis group. Interestingly, based on the percentage of baseline (D0) concentration, REG3E appeared to decrease faster than CRP on D2 and D3, suggesting that REG3E either has a shorter plasma half‐life than CRP or that its production is downregulated more rapidly after treatment of sepsis. On the other hand, no correlation was found between both proteins within the TPLO group, where we only saw an increase in CRP but not REG3E concentrations post‐surgery. Taken together, our findings suggest that CRP is likely a more reliable marker for the current inflammatory state than REG3E in dogs, as it increased more consistently after mild stimulation (TPLO), decreased more reliably after treatment for sepsis, and appeared to be less influenced by other factors such as concurrent renal and pancreatic disease.

Our three study cohorts were comparable in terms of age but not sex. There was a female predominance in the sepsis group, including three entire bitches, all of which presented with pyometra. On the other hand, male intact dogs represented a third of the population in the healthy dogs but were underrepresented in both other groups. A possible sex influence on REG3E concentrations and kinetics has to date not been explored in either people or dogs, and our groups were too small to partition by sex to explore a possible sex influence. Both the high prevalence of male entire dogs and Shepherd breeds in the healthy group is likely due to the inclusion of military service dogs in this group. The inclusion of a significant proportion of working dogs may have influenced the diversity within our group of healthy dogs, as these dogs not only belong to a single breed group but also have a lifestyle that differs considerably from those of typical pet dogs. This is highlighted in our results by the significantly higher REG3E concentration in healthy military working dogs versus healthy pet dogs, as well as the lower interindividual variability of REG3E within the military dog subgroup. Age may have contributed to this difference, as military dogs in this study were on average younger than dogs in other groups. In people, REG1A concentrations have been shown to be highest in infants and decrease with age [36], but no such age effect has to date been examined for REG3E in dogs. A breed effect was considered less likely, as pet Shepherd dogs in both the healthy and the TPLO group had generally low REG3E values, often below the LLoQ. Strenuous exercise has been shown to elevate acute‐phase protein concentrations in dogs, including CRP, haptoglobin, and serum amyloid A [37], but CRP concentrations were below the LLoQ in all healthy dogs in our study, including working dogs, suggesting that this was unlikely to play a role in this cohort.

Our study had other limitations, the most important being the use of samples collected for another kinetic study and leftover convenience samples. Thus, we could not control the exact time points of blood sampling, as these were at the attending clinicians' discretion, depending primarily on clinical factors as well as on the weekday. Furthermore, we did not have measurements at every time point for all dogs, which could have weakened our data pool. By only including dogs with at least three data points, we may have biased our case selection toward clinically less severe sepsis with a favorable survival rate, as dogs succumbing to their disease within 1–2 days were not included. Conversely, dogs which recovered faster after TPLO would have been sent home sooner and would have missed sampling at D2, potentially favoring dogs with delayed recovery in this cohort. As our institution is a referral hospital, many sepsis cases were first seen by a primary care veterinarian, which may have delayed the time between onset of sepsis and the first sampling (D0). Furthermore, some of these dogs were treated with antibiotics among other drugs prior to admission, which was not always reliably documented in the medical records. Therefore, the therapeutic protocol differed between cases, which may have influenced the trajectory of acute‐phase proteins. Additionally, the different sources of sepsis made it impossible to standardize this cohort. Ideally, kinetics of such proteins would be measured after controlled induction of a septic stimulus and measured in closer intervals and over a longer time frame, as described for PCT [10], but such experimental studies with healthy dogs are not feasible in our country for ethical reasons.

In conclusion, REG3E concentrations stayed relatively constant at low levels for 3 days in health and after orthopedic surgery, whereas they were high and decreased progressively over 48–72 h in most dogs with sepsis. However, REG3E appeared to be influenced by factors other than sepsis, and with our study design, CRP seemingly performed better as a marker for inflammation, particularly mild postoperative inflammation. Nonetheless, the value of REG3E as an early indicator of sepsis persistence or recurrence in different clinical scenarios deserves further investigation.

## Funding

The majority of samples used in this project were originally sampled as part of a study supported by a grant from the Swiss Federal Food Safety and Veterinary Office (FSVO Grant No. 1.20.03). Additional financial support was granted by the Faculty Clinical Research Platform of the Vetsuisse Faculty, Bern, Switzerland.

## Conflicts of Interest

The authors declare no conflicts of interest.
